# Top-down modulation of visual action perception: distinct task effects in the action observation network

**DOI:** 10.1007/s00429-025-03042-z

**Published:** 2025-11-10

**Authors:** Aslı Eroğlu, Burcu A. Urgen

**Affiliations:** 1 Max Planck Institute for Biological Cybernetics, Tübingen, Germany; 2 Department of Neuroscience, Bilkent University, Ankara, Turkey; 3 Department of Psychology, Bilkent University, Ankara, Turkey; 4 Aysel Sabuncu Brain Research Center and National Magnetic Resonance Research Center (UMRAM), Bilkent University, Ankara, Turkey

**Keywords:** Action observation, Attention, Top-down modulation, FMRI, Representational similarity analysis, Decoding analysis

## Abstract

Perceiving others’ actions is essential for survival and social interaction. Cognitive neuroscience research has identified a network of brain regions crucial to visual action perception, known as the Action Observation Network (AON), comprising the posterior superior temporal cortex (pSTS), posterior parietal cortex, and premotor cortex. Recent research highlights the importance of integrating top-down processes, such as attention, to gain a deeper understanding of action perception. This study investigates how attention modulates the AON during human action perception. We conducted a two-session fMRI experiment with 27 participants. They viewed eight videos of pushing actions, varying in actor (female vs. male), effector (hand vs. foot), and target (human vs. object). In the first session, participants focused on specific features of the videos (actor, effector, or target). In the second, they passively viewed the videos. From the passive viewing session data, we defined regions of interest (ROIs) in the pSTS, parietal, and premotor cortices for each hemisphere. We then performed model-based representational similarity analysis (RSA) and decoding analysis. RSA results showed that only the task model, among all tested models, exhibited a significant correlation with neural representational similarity matrices (RDMs) across all ROIs, indicating a specific alignment between AON nodes and the ongoing task. Decoding analysis further showed that different task types uniquely affected each AON node, indicating feature- and region-specific interactions. These findings underscore that top-down attentional processes significantly alter neural representations within the AON, highlighting the dynamic interplay between attention and action perception in the brain.

**Supplementary Information:**

The online version contains supplementary material available at 10.1007/s00429-025-03042-z.

## Introduction

Perception of others’ actions is one of the core abilities to navigate and comprehend the external world. Its evolutionary roots extend deep into the animal kingdom, which serves as a vital survival tool, allowing organisms to identify opportunities and threats in their environment. For humans, action perception goes beyond mere survival; it also plays a central role in social interactions. Our capacity to interpret the actions of others has been the cornerstone of effective communication, collaboration, and self-preservation.

**Fig. 1:**
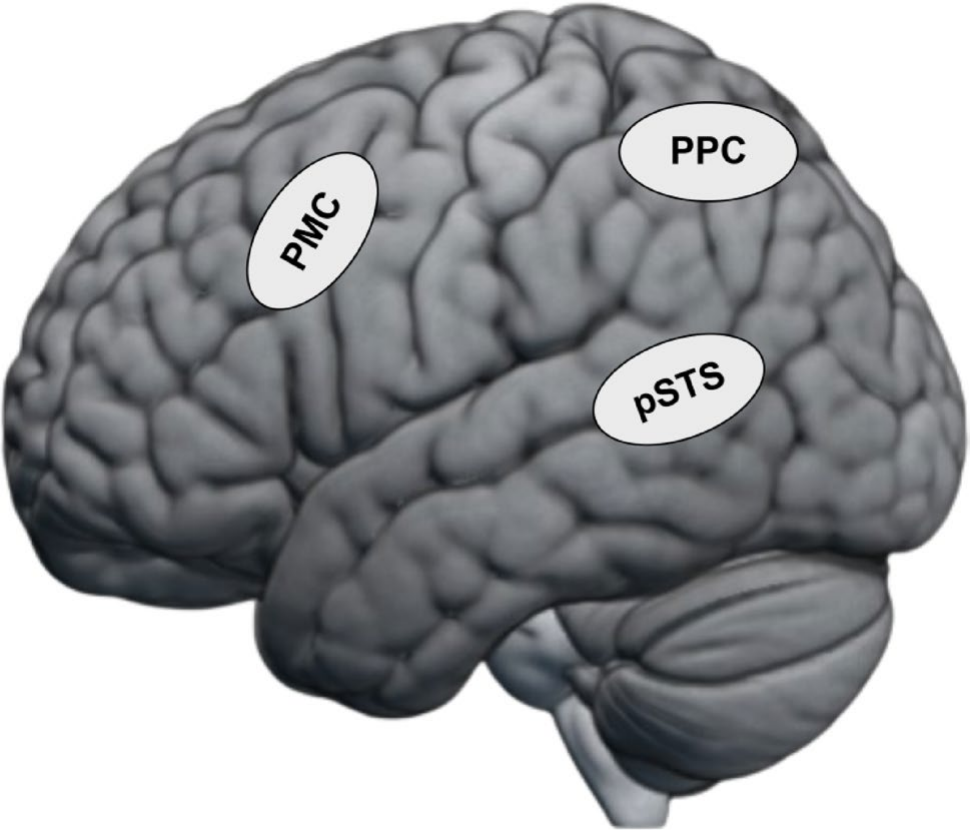
The core regions of the Action Observation Network: posterior superior temporal cortex (pSTS), posterior parietal cortex (PPC), premotor cortex (PMC)

Years of cognitive neuroscience research show that visual perception of actions is supported by a network of regions including the early visual cortex and motion-sensitive regions like MT+, extrastriate body area (EBA) (Grossman and Blake 2002; Jastorff and Orban 2009; Peelen et al. 2006) which then extend into the Action Observation Network (AON) (Caspers et al. 2010; Nelissen et al. 2011; Saygin 2007). This network’s core regions include the posterior superior temporal cortex (pSTS), the posterior parietal cortex (PPC), and the premotor cortex (PMC) (Fig. 1).

The lateral occipitotemporal cortex (LOTC) is a heterogeneous mosaic of overlapping subregions involved in processing bodies, body–object interactions, and social cues (Lesourd et al. 2023). Activity patterns in LOTC integrate signals from more focal regions such as the extrastriate body area and hMT, forming distributed representational spaces that span both perceptual and conceptual dimensions (Kabulska et al. 2024). Multivariate decoding studies show that LOTC codes action categories and higher-order features such as sociality and transitivity, with representations that generalise across actors, effectors, viewpoints, and movement kinematics (Wurm et al. 2017). This evidence has led to proposals that LOTC forms the core of a lateral “what” pathway for action recognition, complementing the dorsal (visuomotor) and ventral (object) streams (Simonelli et al. 2024; Wurm and Caramazza 2022). Within this broader architecture, different LOTC subregions show distinct tuning and lateralisation—for instance, left LOTC/pSTS is more responsive to animacy and gesture meaning, whereas right LOTC/pSTS is more attuned to kinematic features (Simonelli et al. 2024). Among these subregions, the posterior superior temporal sulcus (pSTS) has been studied most extensively for its role in biological motion and social action perception.

In humans, pSTS responds more strongly to biological‑motion displays than to random or object motion and is activated by stories that include incidental descriptions of biological motion (Deen and McCarthy 2010). This region carries information about communicative actions (Kabulska et al. 2024) and is particularly sensitive to social intent and symbolic gestures (Simonelli et al. 2024). Electrophysiological recordings in non‑human primates show that neurons in the upper bank of the STS selectively respond to dynamic actions and can discriminate forward from reversed movements (Vangeneugden et al. 2011). Combined fMRI and tract‑tracing work indicates that these STS regions provide the visual input that is relayed via parietal nodes (PFG/AIP) to premotor cortex (Nelissen et al. 2011). This converging evidence has led many researchers to consider pSTS the entry point of the action‑observation network. Beyond action perception, pSTS also encodes socially relevant cues such as gaze direction (Senju and Johnson 2009) and head orientation (Carlin and Calder 2013). These findings converge to portray pSTS as a hub that integrates visual information for action understanding while simultaneously decoding higher-order social signals (Deen et al. 2015; McMahon and Isik 2024; Wurm et al. 2017). This dual capacity for processing both movement and social meaning underlies its identification as a core component of the recently proposed third visual pathway (Pitcher and Ungerleider 2021).

The PPC is another crucial region within the AON, responsible for encoding higher-level visual and abstract information related to actions and forming a coherent action model. Anatomically, it lies posterior to the primary somatosensory cortex and comprises the superior and inferior parietal lobules separated by the intraparietal sulcus (Vingerhoets 2014). Using inputs from pSTS, the PPC represents interactions between target objects and body parts during observed actions (Jastorff et al. 2010; Urgen et al. 2019). Within this region, the intraparietal sulcus integrates visual input with motor parameters and contributes to representing goals of observed actions rather than low-level kinematics (Hamilton and Grafton 2006). The superior parietal lobule (SPL) contributes to multisensory integration and is consistently recruited across action observation, imitation and execution, alongside the supramarginal gyrus and dorsal premotor cortex, indicating its role in linking perception and movement (Molenberghs et al. 2010). The inferior parietal lobule, which includes the supramarginal gyrus, is thought to store and update body-related information: the left inferior parietal region supports stored hand‑posture representations for planning and predicting object-directed action (van Elk 2014), while the right supramarginal gyrus contributes to proprioceptive sense (Ben-Shabat et al. 2015). Because of its capacity to represent goals, context and body state, the PPC integrates visual representations with abstract features such as intention and contextual relevance, so damage to the parietal region can spare visual perception yet impair understanding of the meaning and appropriateness of actions within a given context (Binder et al. 2017).

The premotor cortex (PMC) is a frontal node of the action‑observation network (AON) that works alongside occipito‑temporal and parietal regions to transform visual information about others’ movements into motor plans (Urgen and Orban 2021). This area contains ventral (PMv) and dorsal (PMd) subdivisions, each with a somatotopic organisation. The premotor cortex -and not the parietal cortex - shows a mapping organized by the effector (i.e., the body part) used in the observed action (Jastorff et al. 2010). The number of fingers used during a grasp is also encoded within the PMC, specifically within PMv (Fabbri et al. 2016).

PMv appears specialised for hand–object interactions and fine finger control. Virtual lesions induced via transcranial‑magnetic‑stimulation demonstrate that perturbing PMv disrupts the grasping phase of precision grip, leading to misplacement of the thumb and index finger and delayed recruitment of intrinsic hand muscles (Davare et al. 2006). During action observation, repetition of the grip component attenuates activity in PMv, supporting the idea that this area encodes the kinematic details of hand–object interactions (Majdandžić et al. 2009). PMd, on the other hand, supports reach‑related movements and higher‑level motor planning. Functional MRI reveals that executed, observed and imagined reaching all activate dorsal premotor cortex (Filimon et al. 2007), and virtual lesions of contralateral PMd disrupt the coupling between grasp and lift phases without altering finger placement (Davare et al. 2006). Moreover, PMd activity decreases when the desired end state of an observed action is repeated, indicating that this region encodes action goals rather than grip kinematics (Majdandžić et al. 2009). Together, these findings demonstrate that the PMC is a crucial part of the AON with its ventral and dorsal subdivisions playing complementary roles in the planning, coordination, and execution of actions.

Over the last few years, several reviews and empirical studies have broadened our understanding of the AON. Kemmerer’s (2021) survey catalogued 22 factors that modulate mirror‑neuron responses during action observation, spanning the properties of the action, the actor, the observer, their relationship and context. Thompson et al. (2019) clarified that “action understanding” comprises distinct processes—action identification, goal identification and intention identification—and concluded that current evidence only supports a contribution of mirror‑neuron areas to low‑level action identification. Recent multivariate fMRI work has shown that, although kinematic, object‑related and semantic aspects of actions elicit partially distinct patterns across occipito‑parietal, ventro‑temporal and lateral occipito‑temporal cortex, most AON regions display overlapping, distributed coding (Simonelli et al. 2024). Moreover, the AON may represent the physics of events in a domain‑general manner: neural codes for human actions and inanimate object events overlap across posterior temporal and frontoparietal cortices (Karakose‑Akbiyik et al. 2023), challenging the view that the AON is restricted to animate action recognition (Wang and Jiang 2023). Finally, resting‑state connectivity analyses reveal that the AON and the mentalizing system form distinct yet interacting networks, with the pSTS and anterior IFG potentially mediating their communication (Simone et al. 2025).

These broader perspectives underscore that action perception is shaped by both bottom‑up sensory representations and top‑down modulatory influences. Neuroimaging studies often explore the AON by passively observing actions; however, this method overlooks the complex interplay between bottom‑up and top‑down processes that are crucial for real‑life perception (Kay et al. 2023). Recent research emphasizes integrating cognitive factors like expectation, prediction, and attention into models of action observation (Kilner et al. 2007; Urgen and Miller 2015; Urgen and Saygin 2020). Attention, in particular, is fundamental for efficiently directing cognitive resources and can enhance neural responses to attended stimuli (Borji & Itti 2012; Cohen and Maunsell 2009). Feature‑based attention modulates neural activity by enhancing attended features and suppressing unattended features, revealing the complexity of attentional mechanisms in perception (Maunsell and Treue 2006; Treue 2003). Incorporating top‑down attention tasks in action observation studies is therefore critical for a comprehensive understanding of how the AON processes actions in realistic contexts.

There are some important studies investigating how the attention mechanisms modulate the AON. One such study conducted by Stehr et al. (2021) investigated how action coding in the pSTS region changed under different attention conditions using functional magnetic resonance imaging (fMRI). Their findings revealed that directing attention to action categories significantly enhanced the distinctiveness and categorization of neural responses within the right pSTS. This suggests that attention influences the overall activity in the pSTS and shapes the way neural populations represent different actions. These findings highlight the significance of top-down processes in shaping the perception.

In an fMRI study conducted by Orban and colleagues (2019), participants observed a set of videos in which an actor manipulated an object while engaging in tasks that involved distinguishing between action categories (pushing or pulling the object), identifying the object’s color, or recognizing the performing actor’s identity. Within the Region of Interest (ROI) analyses, researchers included certain regions, such as the lateral-occipital regions near the pSTS, the parietal cortex, and the premotor cortex. The results suggest that participants’ brain activities varied depending on the attention tasks, even when viewing the same videos. Specifically, when participants engaged in the action discrimination task, activation was observed in the parietal cortex, contrasting with the lack of activation in other discrimination tasks. Additionally, the authors stated that lateral-occipital areas showed activation in a non-task-dependent manner, while the premotor region exhibited task specificity. This suggests that not every attention task equally influences every brain region; instead, different tasks affect brain regions to varying degrees.

Another significant study supporting the idea that top-down attention processes can influence action perception was conducted by Shahdloo et al. (2022). This study addresses the brain’s representation of diverse visual action categories and examines how these representations dynamically change depending on the task performed. Participants watched a series of videos containing numerous visual action categories (e.g., ‘hitting’ or ‘lifting’). They performed a task requiring a covert search for the target action categories while their brain activity was measured using fMRI. In summary, the article demonstrates that a natural visual search for specific action categories influences semantic representations in the brain, causing tuning shifts in neural activity toward the target category within and beyond the AON. This suggests that dynamic attentional mechanisms enhance action perception by effectively allocating neural resources to emphasize task-relevant action categories. This provides insights into how humans perceive others’ actions in dynamic daily life experiences.

As evident from the examination of the limited studies conducted in this field, research on the impact of top-down attention processes on action perception has generally focused on manipulated objects, actors, and action classes. However, it is known that the body parts used during the execution of an action also play a significant role in constructing the meaning of the action. This feature is processed in the premotor cortex and the parietal cortex of the AON (Jastorff et al. 2010), and directed attention to the effector can specifically influence these regions. Furthermore, the target to which the action is directed is another crucial aspect affecting the meaning of the action. For example, when the target is an object, a pushing action is considered a manipulation action. In contrast, if the target is a human, it becomes a social action with a specific emotional value. Despite their relevance, the impact of attention tasks on the perception of specific action features (e.g., actor, effector, target) remains unexplored in the action perception literature. Additionally, studies have not yet directly controlled attention on these features to systematically examine how they interact with one another.

This study investigates the dynamic interaction between attentional processes and the AON across the pSTS, parietal, and premotor cortex. Specifically, we seek to understand how attention modulates these regions during the perception of human actions, building upon prior neuroimaging studies. To achieve this, our methodology involves functionally localizing core AON regions using univariate analysis in a passive viewing session where participants observed the action videos. Subsequently, we apply univariate, representational similarity analysis (RSA) and decoding analysis on the data from active sessions, where participants engage in attention tasks. We aim to investigate whether attention directed towards the actor, effector, or target features of an action differentially modulates activity across the AON. This research may reshape our understanding of action perception, suggesting a more complex interplay between attention and neural processing than previously assumed.

## Materials & methods

### Participants

30 healthy individuals participated in the two-session fMRI experiment. They had either normal or corrected-to-normal vision and were not taking medication for neurological or psychiatric conditions. 3 of the participants were excluded due to excessive motion and technical problems. The remaining 27 participants (25 right-handed, 2 left-handed) included 12 females with a mean age of 24.5 and an age range of 21–31. The study was approved by the Human Research Ethics Committee of Bilkent University, and each participant provided written informed consent and filled out the MRI prescreening form.

### Stimuli, experimental design and procedure

#### Stimuli

**Fig. 2:**
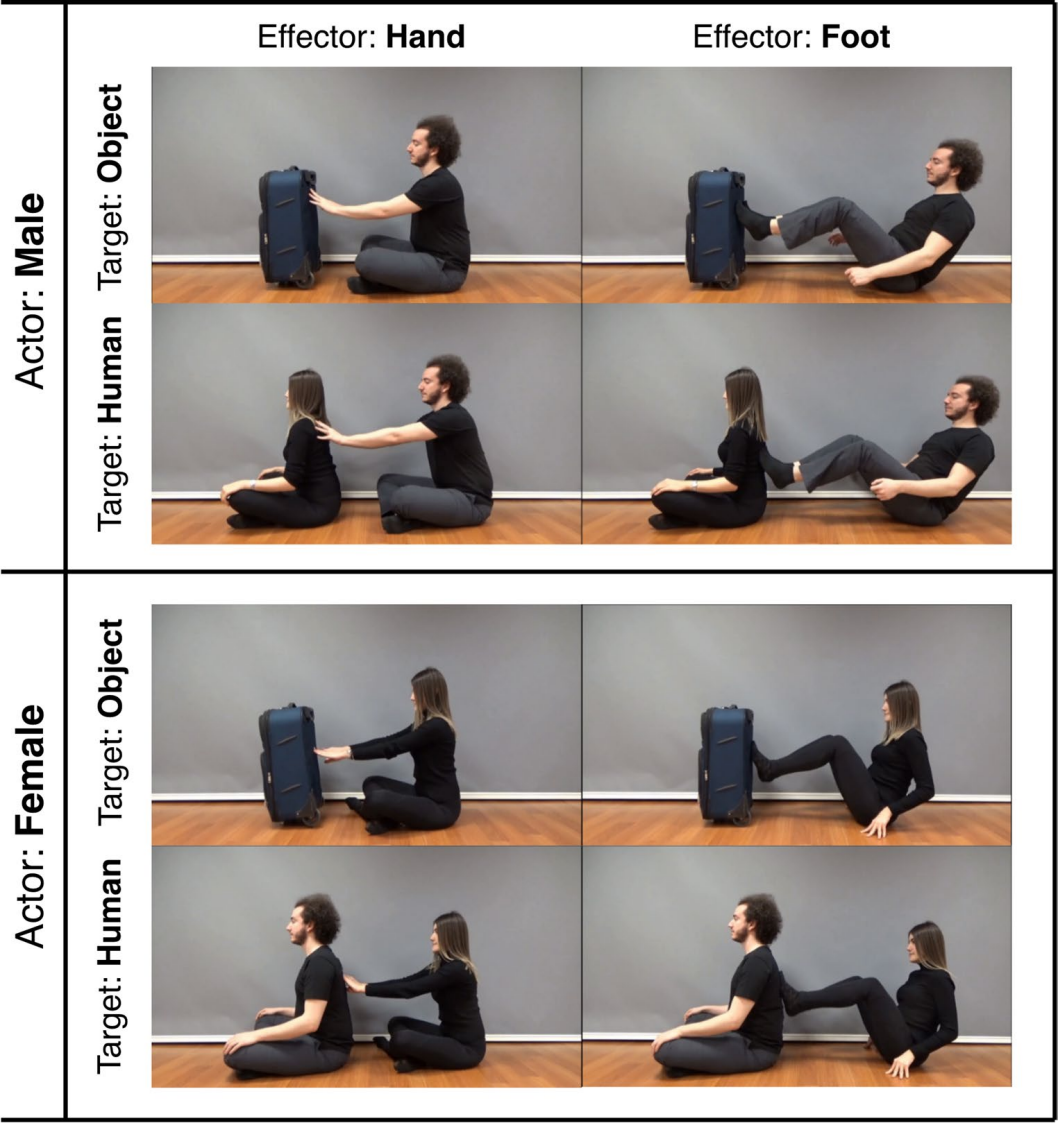
A frame from each of the video stimuli

Eight action videos each lasting 3 s were recorded. All videos depicted a “pushing” action but varied in terms of three parameters: the actor performing the movement, the body part (effector) used for the action, and the target of the action. The actions were performed by two different actors, one female and one male. Each actor performed the pushing action using two different effectors, hand and foot. Finally, the actors directed the pushing action toward two different targets, a human and an object. A frame from each video can be found in Fig. 2.

The videos were recorded with the Sony HDR-CX405 Handycam Video Camera at 50fps and 1280 × 720 resolution. All videos were recorded from the same distance and the actors’ positions were kept fixed and symmetrical. The actors were instructed to minimize any variation in their movements and maintain neutral facial expressions and they practiced the movements before the filming took place/videos were recorded. The clothing of the two actors was chosen to be similar (black top, black pants).

#### Experimental design and setup

The study consisted of two fMRI sessions. The first session was an ‘active’ session where participants watched the video stimuli under task conditions and did evaluations about the video. The second session was a ‘passive’ session where the same stimuli were presented without any task.

The active session was composed of 8 runs and each run lasted approximately 8 min. Each fMRI run included three distinct attention tasks—**Actor**,** Effector**, and **Target**—each referred to as a task block (Fig. 2). Each block type was presented twice within each run, resulting in a total of 6 block presentations. Three attention blocks were grouped together to form one set, and each set was shown twice, with the order of blocks randomized within each set.

**Fig. 3:**
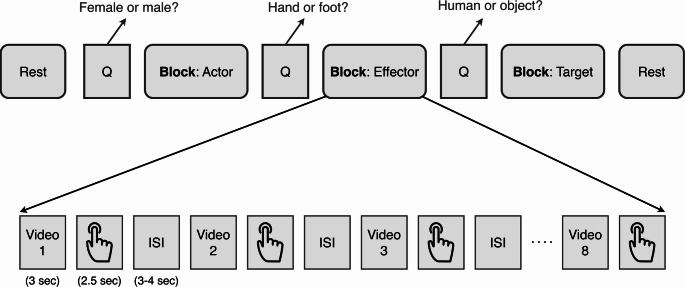
Experimental design for the active session, showing one set (top) and one block (bottom). ISI: Interstimulus interval

Before each block, participants were presented with an instruction screen (4s) indicating the task they would perform in that block (Fig. 3, top section, ‘Question’ screen). Based on the question presented, participants evaluated the videos during the block using a binary button after they watched the video. In the Effector block, they evaluated whether the action was performed with the hand or the foot. In the Actor block, they evaluated whether the actor was female or male, and in the Target block, they evaluated whether the action was directed towards a human or an object. Before and after each set in each run, there was a 12-second rest block.

Within each block, 8 different video stimuli were randomly presented (Fig. 3, bottom section). After each video, participants were given 2.5 s to answer the question using either their left or right thumb on a binary button box. Following this, interstimulus intervals (ISI) between 3 and 4 s were shown, during which participants fixated on the fixation cross. The average duration of the ISIs within each block was 3.5 s.

A practice session was conducted to ensure participants’ understanding of the experiment. In this mini-experiment, each block was shown once. Instead of displaying all eight videos, two videos were carefully selected to be shown in the blocks. These two chosen videos cover all the different variations (one video consisted of the “female, hand, and object” condition, while the other is from the “male, foot, and human” condition).

In the passive session, there were 4 runs, each lasting approximately 6 min. The participants viewed the same set of stimuli without any required response or task to perform. In each run, they passively viewed a block of 8 videos, with each block repeated six times. This resulted in each video being presented a total of 24 times. Data from this session was used to examine the effects of attention tasks, as well as to extract ROIs within the AON to perform further analysis.

Video stimuli in the MRI machine were presented to the participants through the TELEMED screen, which they viewed through a mirror mounted on the head coil (~ 10 cm distance between the mirror and eyes). The TELEMED screen had a refresh rate of 60 Hz and a resolution of 1920 × 1080. The videos were displayed on the screen in a size of 550 × 340 pixels, with a uniform gray background. Stimulus presentation and response collection codes were written using the PsychoPy library and custom functions in Python.

### MRI data acquisition

Participants’ brain activity was recorded using a 32-channel head coil on a 3 T Siemens TimTrio MRI scanner at the National Magnetic Resonance Research Center (UMRAM) at Bilkent University. Soft cushions made of materials that minimize head movement were placed under the participants’ heads, around their necks, and under their legs for support. Earplugs were also provided to reduce exposure to the high noise levels in the MRI machine.

Prior to the functional scans, high-resolution T1-weighted structural scans of participants’ brains were acquired for both experiments (TR = 2600 ms, TE = 2.92 ms, flip angle = 12°, FoV read = 256 mm, phase FoV=%87.5, 176 slices, and 1 × 1 × 1 mm^3^ resolution). Then, for the active session, 168 functional scans and for the passive session 129 functional scans were obtained using gradient echo-planar imaging (TR = 3000 ms, TE = 22 ms, flip angle = 90°, 64 × 64 matrix, FoV read = 192 mm, 43 slices with a thickness of 2.5 mm and a resolution of 3 × 3 × 2.5 mm^3^).

#### Anatomical and functional data preprocessing via fMRIPrep

Results included in this manuscript came from preprocessing performed using fMRIPrep 21.0.1 (Esteban et al. 2019), a Nipype (Gorgolewski et al. 2011) based tool. Each T1w (T1-weighted) volume was corrected for INU (intensity non-uniformity) using N4BiasFieldCorrection v2.1.0 (Gorgolewski et al. 2017) and skull-stripped using antsBrainExtraction.sh v2.1.0 (using the OASIS template). Brain surfaces were reconstructed using recon-all from FreeSurfer v6.0.0 (Dale et al. 1999), and the brain mask estimated previously was refined with a custom variation of the method to reconcile ANTs-derived and FreeSurfer-derived segmentations of the cortical gray-matter of Mindboggle (Klein et al. 2017). Spatial normalization to the ICBM 152 Nonlinear Asymmetrical template version 2009c (Fonov et al. 2009) was performed through nonlinear registration with the antsRegistration tool of ANTs v2.1.0 (Avants et al. 2008), using brain-extracted versions of both T1w volume and template. Brain tissue segmentation of cerebrospinal fluid (CSF), white-matter (WM) and gray-matter (GM) was performed on the brain-extracted T1w using fast (Zhang et al. 2001) (FSL v5.0.9).

Functional data was slice time corrected using 3dTshift from AFNI v16.2.07 (Cox 1996) and motion corrected using mcflirt (FSL v5.0.9 (Jenkinson et al. 2002). This was followed by co-registration to the corresponding T1w using boundary-based registration (Greve and Fischl 2009) with 9 degrees of freedom, using bbregister (FreeSurfer v6.0.0). Motion correcting transformations, BOLD-to-T1w transformation and T1w-to-template (MNI) warp were concatenated and applied in a single step using antsApplyTransforms (ANTs v2.1.0) using Lanczos interpolation.

Physiological noise regressors were extracted applying CompCor (Behzadi et al. 2007). Principal components were estimated for the two CompCor variants: temporal (tCompCor) and anatomical (aCompCor). A mask to exclude signal with cortical origin was obtained by eroding the brain mask, ensuring it only contained subcortical structures. Six tCompCor components were then calculated including only the top 5% variable voxels within that subcortical mask. For aCompCor, six components were calculated within the intersection of the subcortical mask and the union of CSF and WM masks calculated in T1w space, after their projection to the native space of each functional run. Frame-wise displacement (Power et al. 2014) was calculated for each functional run using the implementation of Nipype.

Many internal operations of fMRIPrep use Nilearn (Abraham et al. 2014), principally within the BOLD-processing workflow. For more details of the pipeline see http://fmriprep.readthedocs.io/en/latest/workflows.html.

### Behavioral analysis

Behavioral analysis of the active session focused on reaction times and accuracy. A one-way repeated measures analysis of variance (ANOVA) was conducted across the three tasks to assess potential differences in task difficulty.

### Univariate analysis and activation maps

After preprocessing, we first conducted a univariate analysis using the SPM12 software. The analysis employed the general linear model (GLM) in both active and passive sessions.

In the passive session, the GLM included the following regressors: action movies, rest, instruction, ISI, and 6 head movement regressors extracted from preprocessing. Functional scans from the passive session were smoothed with a full width at half maximum (FWHM) = 4 mm kernel. Then, we contrasted the action movies condition with the rest condition. The resulting contrast map was used to establish the ROIs for further analysis. Six distinct ROIs in the AON, which are pSTS, parietal, and premotor regions in both hemispheres, were extracted.

In the active session’s univariate analysis, each task condition was defined as a distinct variable. These variables included 3 attention task blocks, rest, instruction, ISI, response duration, and head movement constants for each run. For further analysis in the active session, two more GLMs were run based on the analysis methods that will be conducted. For the RSA, each video within each task was defined as a distinct variable. For decoding analysis, each trial was treated as a separate variable.

Following the estimation of the beta values in GLM, contrast analysis was performed to compare activation differences among conditions. The beta images are smoothed with a 4 mm FWHM kernel before applying group level analysis for the univariate analysis (Beta images were not smoothed for the RSA and decoding analysis). We contrasted the Active > Rest and Passive > Rest conditions per subject (sessions modeled separately), then entered the resulting contrast images as pairs into a second-level paired t-test in SPM25 (Tierney et al. 2025) to assess Active vs. Passive. Whole-brain results used a cluster-forming threshold of *p* < 0.001 with FWE correction at *p* < 0.05.

We also supplied additional analysis in the Supplementary Material. For the active session, we contrasted each attention block with the rest block (Figure S1). Also, to examine the overall task effect and enable a comparison with the contrast image from the passive session, we contrasted the ‘all tasks’ condition with the ‘rest’ condition (Figure S2).

### Identification of rois

**Fig. 4:**
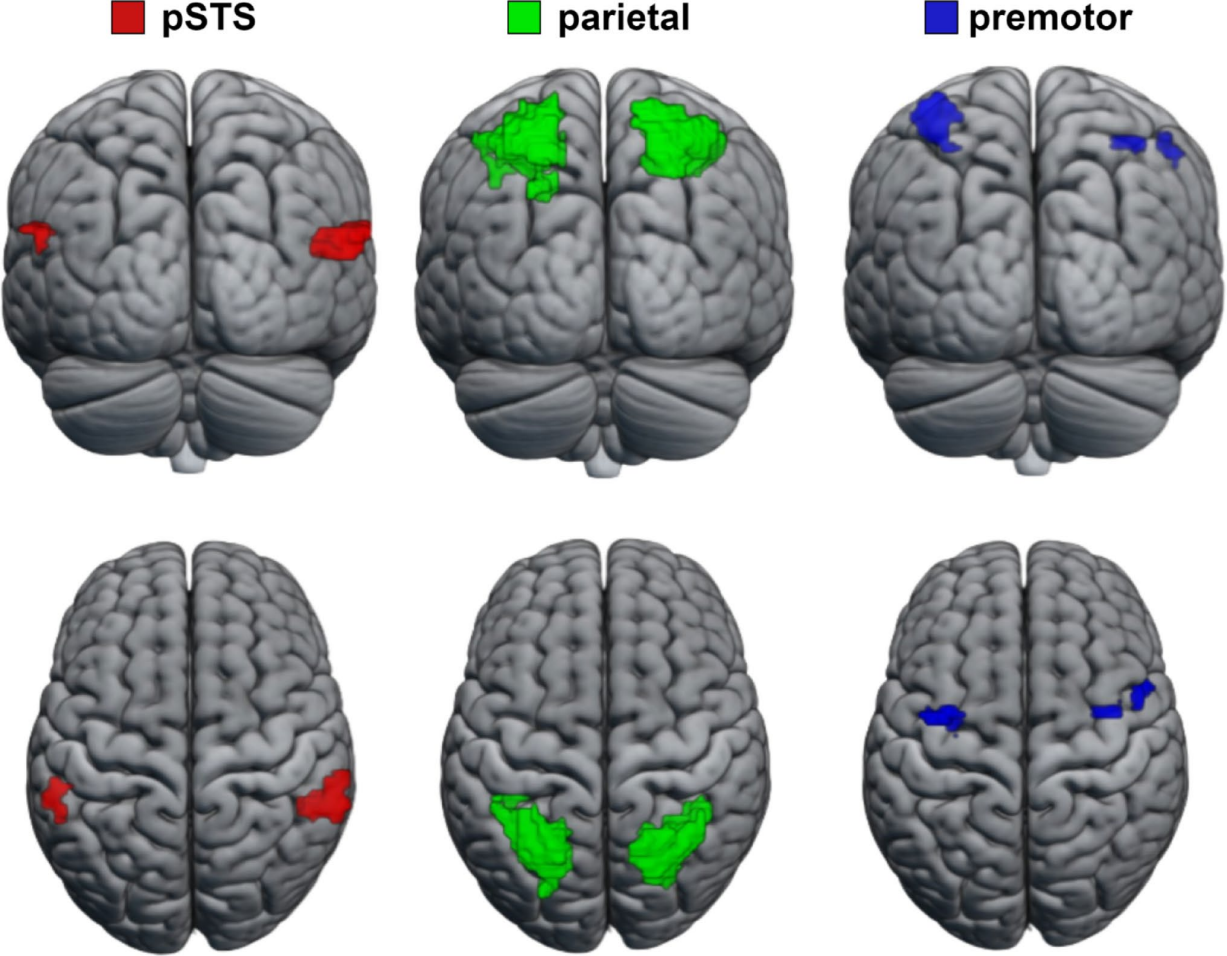
ROIs derived from the group-level analysis

For RSA and decoding analysis, the ROIs were determined using the data from the passive session. We calculated the group average of the movies and rest contrast with a p-value threshold of *p* < 0.001 in order to include the three levels of the AON (uncorrected). When selecting the borders of the ROIs (pSTS, parietal, and premotor cortices), we used specific brain regions from the automated anatomical atlas (AAL) (Rolls et al. 2020) to mask the results. Then, we selected activated voxels within the atlas region. The brain regions used for masking are Temporal_Sup for pSTS, the union of Parietal_Inf and Parietal_Sup for the parietal cortex, and Precentral for the premotor cortex. The saved voxels for the premotor regions were found within the expected premotor activation based on previous studies (Ferri et al. 2015). The coordinates and cluster numbers of the extracted ROIs can be found in Table 1; Fig. 4.

**Table 1:** Cluster number, peak thresholds, and peak coordinates of the defined rois

Region of interest	Left hemisphere			Right hemisphere		
	Cluster number	Peak threshold	Peak coordinates	Cluster number	Peak threshold	Peak coordinates
pSTS	k = 27	t = 4.98	−54 −46 19	k = 114	t = 6.47	54 − 42 19
parietal	k = 440	t = 9.11	−28 −58 59	k = 459	t = 8.19	36–54 56
premotor	k = 68	t = 5.95	−36 −4 52	k = 17 k = 22	t = 5.14 t = 4.92	38 − 4 49 50 2 46

### Representational similarity analysis

We conducted a model-based representational similarity analysis to explore the representational content within the core nodes of the AON (pSTS, parietal, and premotor cortex). The analysis was carried out using the RSA Toolbox (Nili et al. 2014), with some modifications made to the built-in scripts. Contrast images were generated for all 24 regressors (3 tasks x 8 videos) in comparison to the rest. The thresholded SPM images (spmTs) resulting from this analysis were employed as inputs for the RSA.

**Fig. 5:**
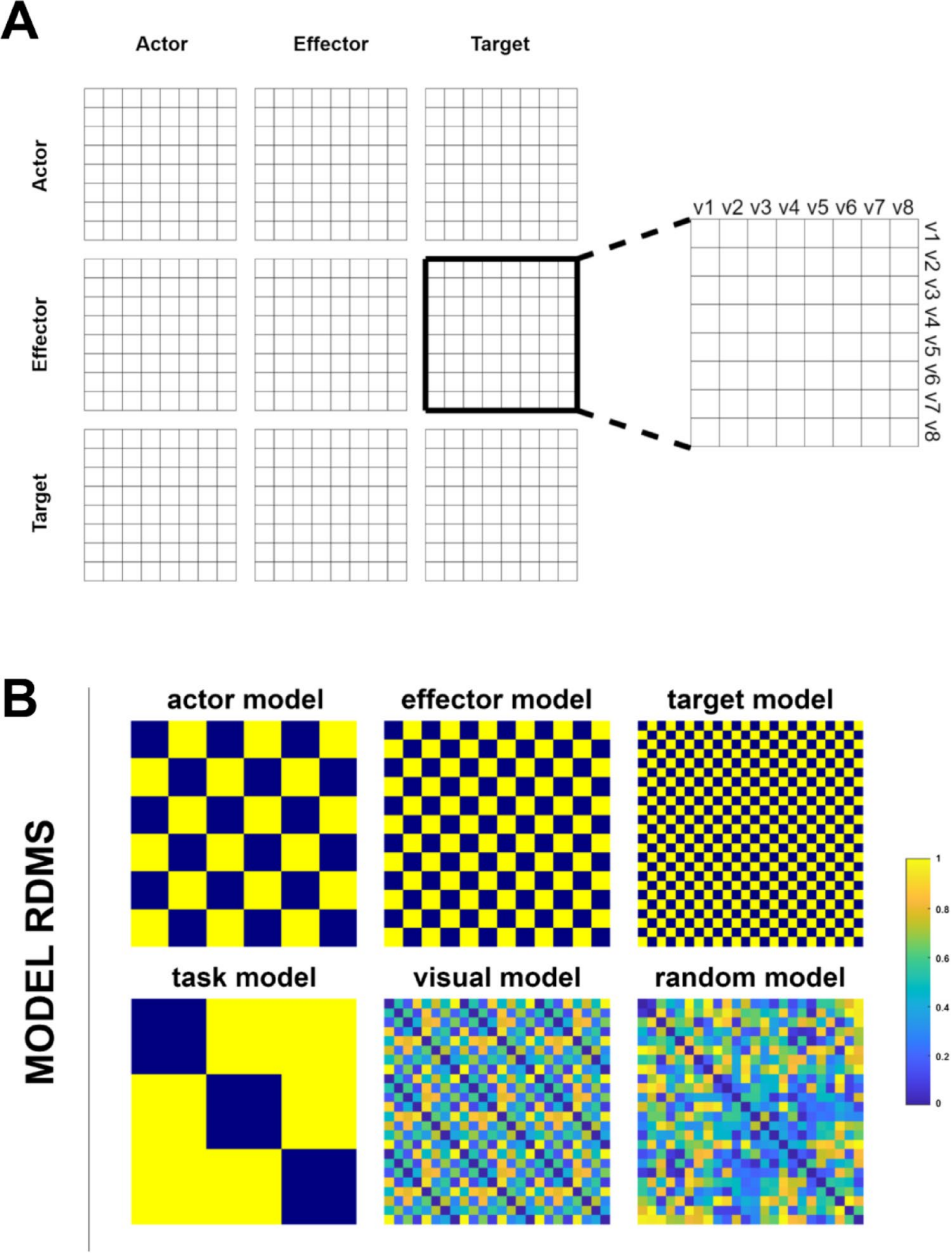
**A** The structure of the model RDMs presents a 24 × 24 matrix, including 8 videos within each of the 3 tasks. **B** Six model RDMs, to be compared with neural RDMs for each ROI. The actor, effector, and target models represent the features of the videos. The task model includes the attention blocks in the active session. The visual model illustrates how videos are similar based on their low-level visual features. The random model is generated using random numbers between 0 and 1 to serve as a control

We generated six distinct models to examine the correlations between model and neural representational dissimilarity matrices (RDMs). The first three models covered the actor, effector, and target features of the videos. These models were binary matrices, with a value of 1 indicating dissimilarity and 0 indicating similarity. In the Actor model, the representation of same-gender pairs (female-female and male-male) is encoded as 0, while a mixed-gender pair (female-male) is represented as 1. In the Effector model, same-limb pairs (hand-hand and foot-foot) are denoted as 0, and a mixed-limb pair (hand-foot) is denoted as 1. Finally, in the Target model, identical features, such as object-object and human-human pairs, are represented as 0, while different features are represented as 1. The fourth model was the task model, characterized by a binary RDM again. In this case, identical task values (Actor-Actor, Effector-Effector, and Target-Target) correspond to 0, while different task values (Actor-Effector, Actor-Target, Effector-Target) were denoted as 1. The next model, referred to as the visual model, covered the visual similarities of the eight videos. We employed Matlab’s structural similarity function (ssimval) to assess video similarities. This function considers the luminance, contrast, and structure values of the images, and we performed the averaging over all frames for each video. The resulting output values range from 0 to 1, with 1 indicating complete dissimilarity and 0 indicating perfect similarity. Lastly, we introduced a random model to serve as a control model for the analysis. The structure of model RDMs (A) and a visual representation of models (B) can be found in Fig. 5.

### Decoding analysis

To distinguish neural activity within the ROIs, we employed a decoding analysis for both active and passive sessions using the Decoding Toolbox (Hebart et al. 2015). With this analysis, our primary objective was to identify and characterize distinct features within ROIs under different task conditions. We aimed to classify actor (female vs. male), effector (hand vs. foot), and target (human vs. object) features.

For the active session, binary classifications were carried out independently in each hemisphere (left and right), for each ROI (pSTS, parietal, premotor), and across all tasks (actor, effector, target), yielding a total of 54 distinct decoding analyses. The beta images from GLM analysis created for each trial separately were used as inputs for our classification algorithms. A linear support vector machine (LibSVM) (Chang and Lin 2011) was implemented with a fixed cost parameter of c = 1. To validate the robustness of our results, a leave-one-run-out cross-validation procedure was employed.

For the passive session, we independently decoded features (female vs. male, hand vs. foot, and human vs. object) for each hemisphere (left and right) and each ROI (pSTS, parietal, and premotor). This resulted in a total of 18 distinct analyses. The procedure was identical to that used for the active session. To avoid introducing statistical differences between active and passive sessions, an equal number of trials from the passive session were used for the decoding analysis.

We conducted a permutation test to determine whether decoding accuracy values were significantly above chance (50%). For each condition, the observed mean accuracy minus chance was compared to a null distribution generated by randomly flipping the signs of the values, assuming symmetry around zero. A one-tailed p-value was calculated as the proportion of null means greater than or equal to the observed mean. To account for multiple comparisons, we applied the Benjamini-Hochberg correction to control the false discovery rate.

Finally, we conducted a 2 (Hemisphere) × 3 (ROI) × 3 (Task) × 3 (Feature) repeated-measures ANOVA using the decoding accuracy values from the active sessions.

## Results

### Behavioral results

Behavioral results were examined to check whether the participants understood the task and if there was a difference between the tasks in terms of difficulty. The average accuracy rate for all tasks was 95.5% (sd=+−7.9%) across participants. For the actor task, the accuracy was 95.3% (sd =+- 7.6%), for the effector task, it was 95.9% (sd =+−8.3%), and for the target task, it was 95.5% (sd=+−8.1%). A one-way ANOVA was performed to compare the accuracy rates of the tasks. The results indicate no statistically significant differences among these tasks (F(2, 78) = 0.04, *p* = 0.964).

For the response time analysis, the false answers were excluded. The average response time for all tasks was 0.80 s (sd=+−0.18 s) across participants. For the actor task, the average response time was 0.81s (sd =-+ 0.20), for the effector task, it was 0.78s (sd =+−0.18), and for the target task, it was 0.81 (sd=+−0.17). A one-way ANOVA was performed to compare the response times of the tasks. The results indicate no statistically significant differences among these tasks (F(2, 78) = 0.21, *p* = 0.812).

### Univariate analysis and activation maps

#### Data cleaning

One was excluded from the 30 participants who initially participated in the study due to technical problems. Motion regressors were derived from the preprocessing outcome for the remaining 29 participants. The motion exclusion criteria for each run were defined as either having a total movement of one voxel size or half-voxel size spikes. Two participants were entirely excluded due to excessive head motion observed during more than half of the total runs. Additionally, for the first session (active session), one run from three participants and two runs from two participants were removed from the analysis. For the second session (passive session), one run from one participant was also excluded from the analysis.

#### Univariate results

**Fig. 6:**
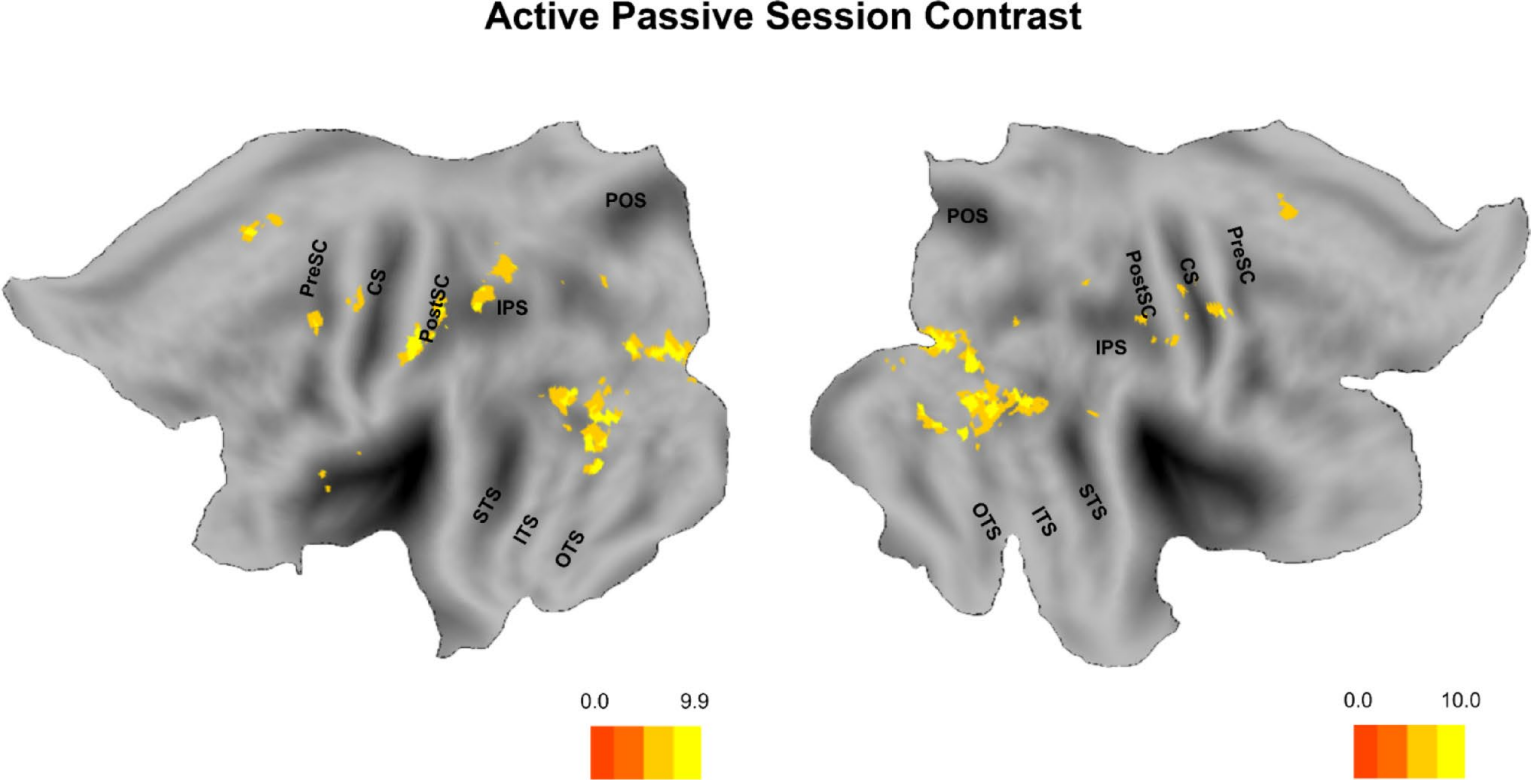
Active vs. Passive (paired t-test). Whole-brain second-level comparison of sessions modeled separately. For each subject, Active > Rest and Passive > Rest contrasts (SPM25; smoothed 4-mm FWHM) were entered as pairs. Maps thresholded at cluster-defining *p* < 0.001; clusters FWE-corrected at *p* < 0.05. Results are displayed on a cortical flat map (Caret5). Anatomical landmarks in black: PreCS (precentral sulcus), CS (central sulcus), PostCS (postcentral sulcus), IPS (intraparietal sulcus), POS (parieto-occipital sulcus), STS (superior temporal sulcus), ITS (inferior temporal sulcus), OTS (occipito-temporal sulcus)

In the paired *t*-test contrasting the active session against the passive session, we observed enhanced activation in a set of regions consistently present in both hemispheres. At a significance threshold of familywise error (FWE) corrected *p* < 0.05, these included early visual areas (V2, V3), the MT cluster and middle temporal gyrus (MTG), and the occipito-temporal sulcus (OTS). Several of these regions are located within the lateral occipito-temporal cortex (LOTC), and the LOTC as a whole exhibited significant activation. Within the posterior parietal cortex, significant clusters were found in the dorsal intraparietal sulcus anterior region (DIPSA) and the putative human anterior intraparietal sulcus (phAIP), along with somatosensory regions BA2 and BA3. Additional activations were detected in dorsal and ventral premotor cortices (PMd, PMv). (Fig. 6).

### RSA results

**Fig. 7:**
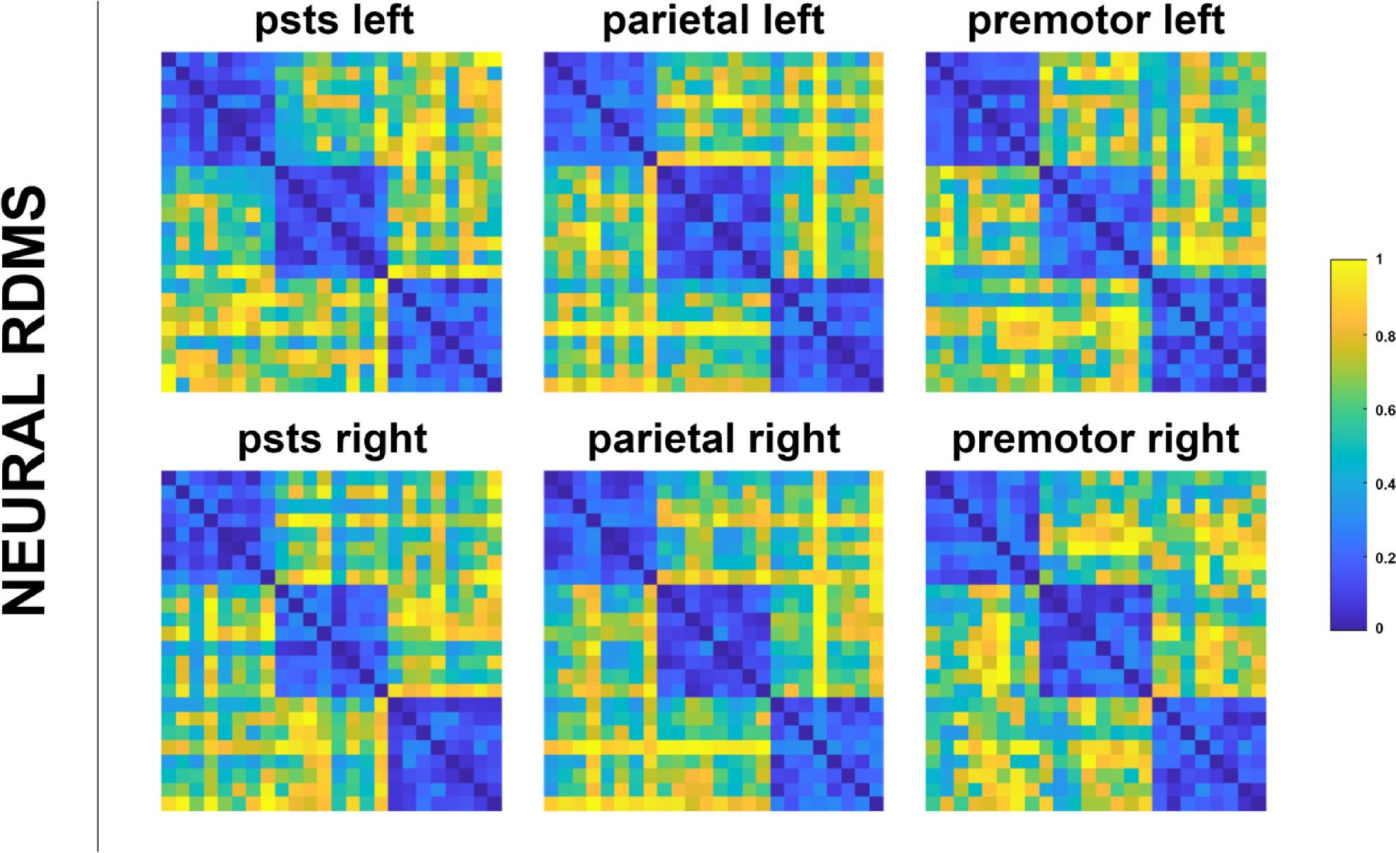
Neural RDMs averaged over all subjects. Each dissimilarity matrix (24 × 24) is separately rank transformed and scaled into [0,1]

**Fig. 8:**
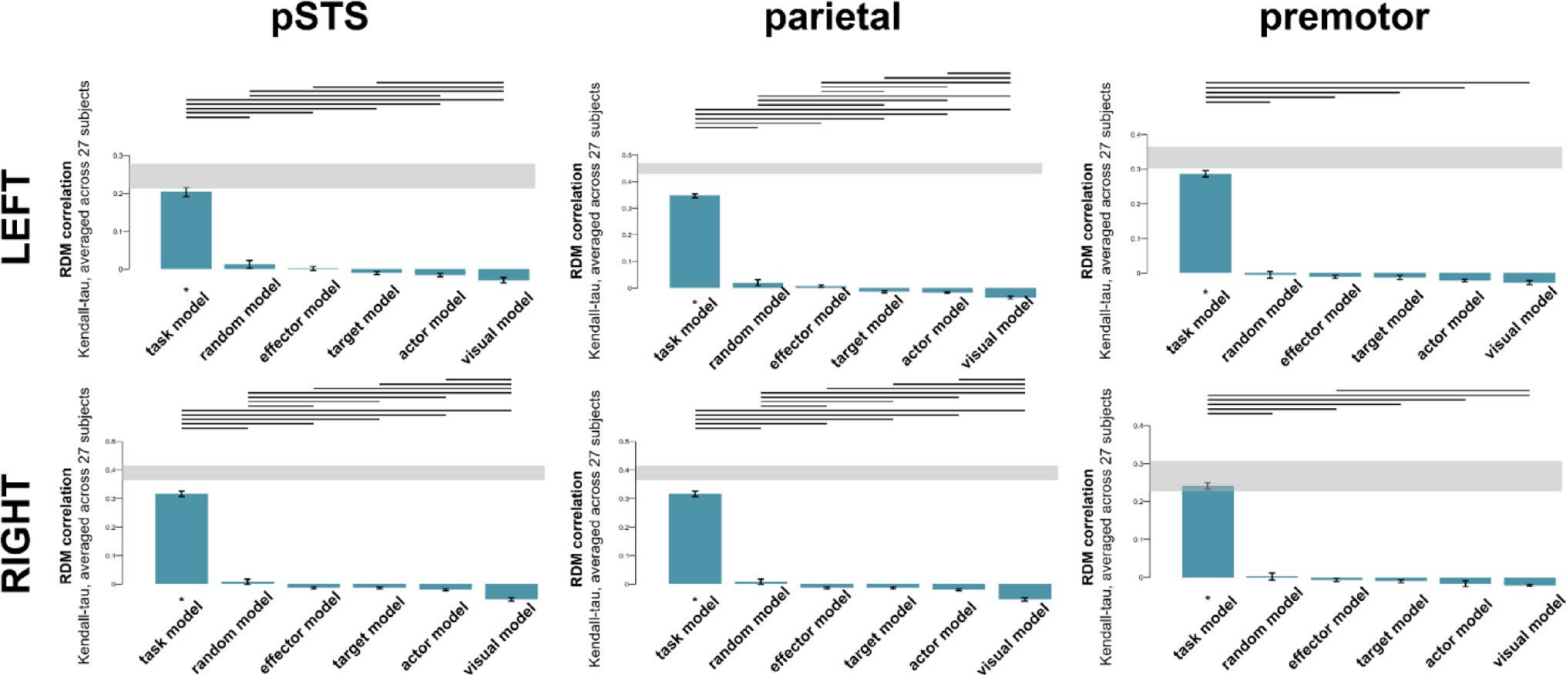
Comparison of neural RDMs and model RDMs. Gray bands over the graphs indicatethe noise ceiling, horizontal black lines indicate significant differences between models, and (*) indicates significant similarity to the ROI RDM at p<0.05 FDR corrected level. Error barsindicate the standard error

Neural RDMs were generated by using a correlation distance measure between each variable’s spmT for each ROI by averaging data from all subjects (Fig. 7). Then, neural RDMs were compared with model RDMs. This comparison was carried out using a one-sided Wilcoxon signed-rank random-effects test corrected for multiple comparisons with false discovery rate (FDR) at a threshold of *p* < 0.05, employing Kendall’s τ as the correlation metric. The upper and lower bounds of the noise ceiling were determined by estimating a hypothetical model with the highest possible average correlation to the reference RDM, once again utilizing Kendall’s τ. Neural and model RDM comparisons are shown in Fig. 8.

For all ROIs, only the task model was found to be significantly correlated with all neural RDMs (*p* < 0.05, FDR corrected); none of the other models were significantly correlated with any ROI. Also, in the right premotor, the task model’s Kendall’s τ value (0.2413) reached the noise ceiling (0.2266, 0.3068). Kendall’s τ and noise ceiling values for all ROIs and models can be found in Table 2.

Figure 8. Comparison of neural RDMs and model RDMs. Gray bands over the graphs indicate the noise ceiling, horizontal black lines indicate significant differences between models, and (*) indicates significant similarity to the ROI RDM at *p* < 0.05 FDR corrected level. Error bars indicate the standard error.

**Table 2:** Statistical inference of comparison of neural RDMs and model RDMs

	Actor Model		Effector Model		Target Model		Task Model		Visual Model		Random Model		
ROI	Kendall’s τ	p	Kendall’s τ	p	Kendall’s τ	p	Kendall’s τ	p	Kendall’s τ	p	Kendall’s τ	p	Noise Ceiling
pSTS left	−0.0152	1.00	0.0020	0.43	−0.0098	0.99	**0.2038**	**0.00**	−0.0291	1.00	0.0126	0.08	(00.2118,0.2784)
pSTS right	−0.0199	1.00	−0.0133	1.00	−0.0135	1.00	**0.3171**	**0.00**	−0.0537	1.00	0.0084	0.11	(0.3642,0.4157)
parietal left	−0.0174	1.00	0.0074	0.13	−0.0138	1.00	**0.3470**	**0.00**	−0.0362	1.00	0.0200	0.06	(0.4289,0.4691)
parietal right	−0.0180	1.00	0.0031	0.66	−0.0063	0.99	**0.3122**	**0.00**	−0.0321	1.00	0.0094	0.13	(0.3816,0.4261)
premotor left	−0.0216	1.00	−0.0113	1.00	−0.0124	0.99	**0.2870**	**0.00**	−0.0277	1.00	−0.0049	0.69	(0.3015,0.3654)
premotor right	−0.0211	1.00	−0.0068	0.92	−0.0101	0.99	**0.2413**	**0.00**	−0.0165	0.98	0.0025	0.42	**(0.2266**,**0.3068)**

Kendall’s τ, spearman’s ρ, and noise ceiling values are calculated. Bold values denote statistically significant results at p < 0.05

### Decoding analysis results

**Fig. 9:**
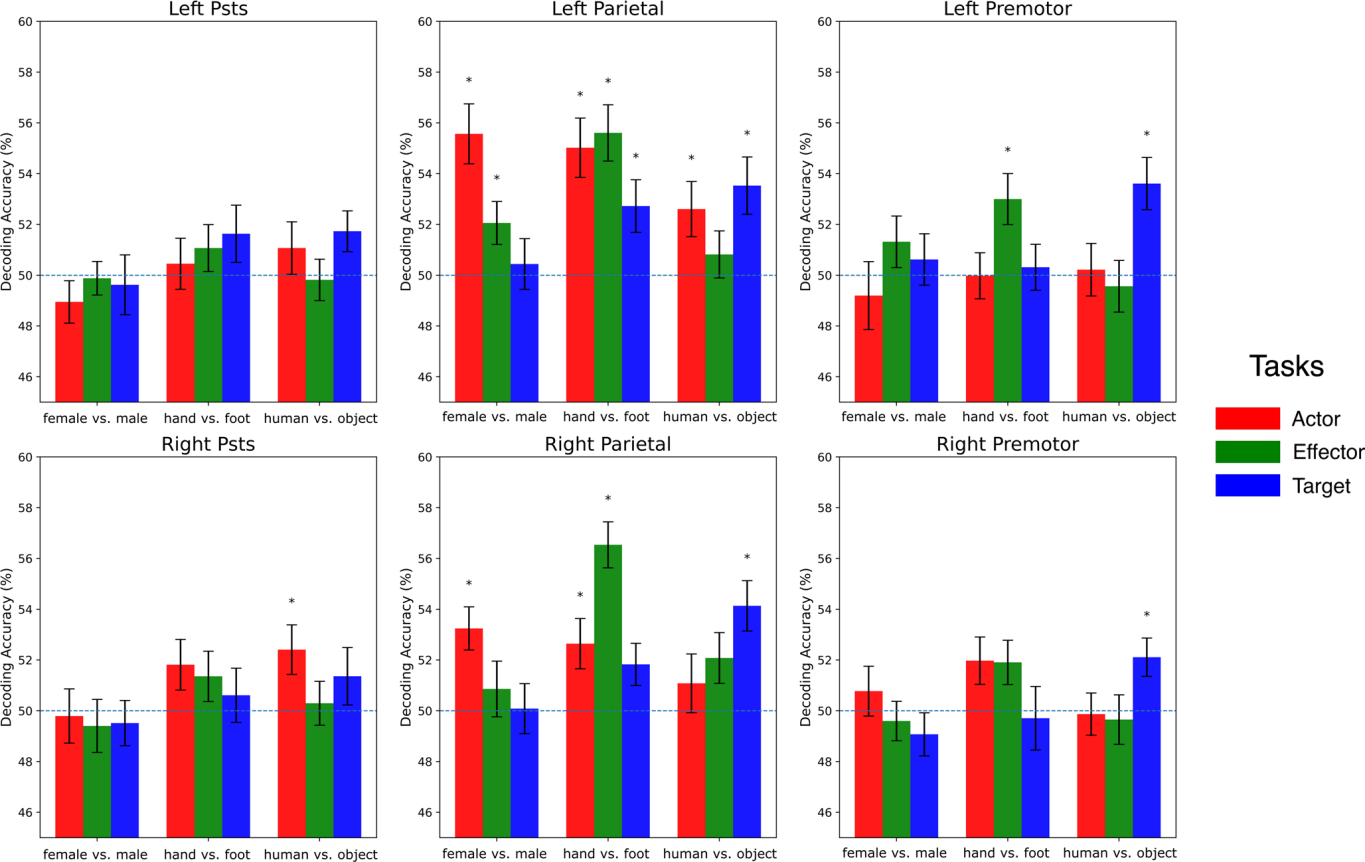
Accuracy values from the decoding analysis for the active session (mean ± SEM across subjects). Significantly successful decodings after FDR correction are marked with “*”

We decoded the features ‘female vs. male,’ ‘hand vs. foot,’ and ‘human vs. object’ for both active and passive sessions. Linear support vector machine classifiers were trained separately for each feature combination. To evaluate decoding accuracy, permutation tests were conducted by comparing the decoding results from 27 subjects against the chance level (50%). Figure 8 shows the accuracy values for the active session, while Fig. 9 shows the accuracy values for the passive session.

For the active session, the pSTS successfully decoded the target of the action (human vs. object) during the actor task in the right hemisphere. None of the other features were successfully decoded in the pSTS under any task condition. On the other hand, the parietal cortex successfully decoded all features under various task conditions, with some slight hemispheric differences. The actor of the action (female vs. male) was successfully decoded during the actor task in both hemispheres and during the effector task in the left hemisphere. The effector of the action (hand vs. foot) was successfully decoded in both hemispheres across almost all task conditions, except during the target task in the right hemisphere. The target of the action (human vs. object) was successfully decoded during the actor and target tasks in the left hemisphere and during the target task in the right hemisphere. In the premotor cortex, the actor of the action (female vs. male) was not decoded under any task condition. However, the effector of the action (hand vs. foot) was successfully decoded during the effector task in the left hemisphere. Additionally, the target of the action (human vs. object) was successfully decoded during the target task in both hemispheres.

For the passive session, none of the features were successfully decoded in the pSTS of either hemisphere. In the parietal cortex of both hemispheres, the effector of the action (hand vs. foot) was successfully decoded, while the other features were not. In the left premotor cortex, the target of the action (human vs. object) was successfully decoded, whereas the other features were not. In the right premotor cortex, no features were successfully decoded, with all decoding accuracies remaining around chance level.

Detailed accuracy-minus-chance values, p-values, and FDR-corrected p-values for each decoding analysis are provided in Supplementary Materials Table 1 (active session) and Supplementary Materials Table 2 (passive session).

**Fig. 10:**
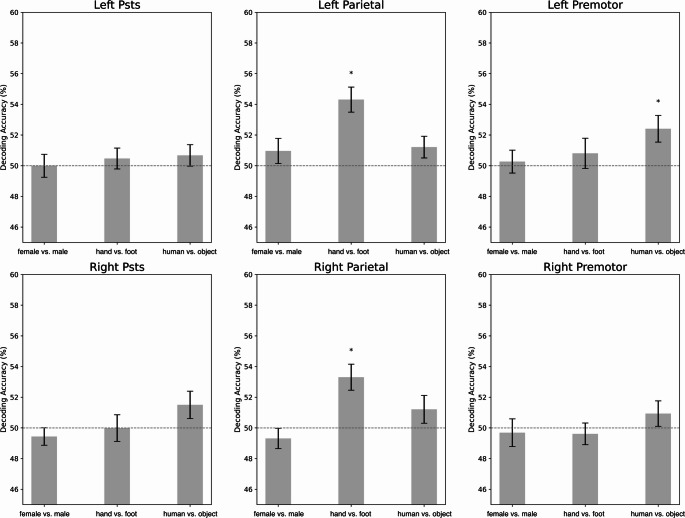
Accuracy values from the decoding analysis for the passive session (mean ± SEM across subjects). Significantly successful decodings after FDR correction are marked with “*”

In order to examine the effect of factors on decoding accuracy and the differences between the conditions, we performed a 2 (Hemisphere) x 3 (ROI) x 3 (Task) x 3 (Feature) repeated measures ANOVA. The statistics for the main effects and interactions are summarized in Table 3.

**Table 3:** Results of the repeated measures ANOVA on decoding accuracy

2 (Hemispheres) x 3 (ROI) x 3 (Task) x 3 (Features) Repeated Measures ANOVA Results			
Cases	F value	P value	η²
hemi	1.433	0.242	–
**roi**	**48.236**	**< 0.001**	**0.038**
task	0.130	0.879	–
**feature**	**7.003**	**0.002**	**0.015**
hemi * roi	0.905	0.411	–
hemi * task	0.526	0.594	–
roi * task	1.728	0.149	–
hemi * feature	0.581	0.563	–
roi * feature	1.535	0.197	–
task * feature	**6.702**	**< 0.001**	**0.024**
hemi * roi * task	**3.443**	**0.011**	**0.007**
hemi * roi * feature	0.332	0.856	–
hemi * task * feature	0.485	0.747	–
roi * task * feature	**2.325**	**0.021**	**0.012**
hemi * roi * task * feature	0.324	0.956	–

Significant results are highlighted in bold

While there was no effect of hemisphere and task (See Table 3 for statistics), there was a main effect of feature (F = 7.003, p = 0.002, η²=0.015) and ROI (F = 48.236, p < 0.001, η²=0.038). Post-hoc tests showed that ‘hand vs. foot’ decoding accuracy was significantly higher than ‘female vs. male’ (MD = 1.566, t = 3.731, p = 0.001 Bonferroni-corrected), with no other significant comparison results for the feature. For ROI, the parietal cortex’s decoding accuracy was significantly higher than the pSTS region (MD = 2.227, t = 8.684, p < 0.001 Bonferroni-corrected) and premotor cortex (MD = 2.133, t = 8.316, p < 0.001 Bonferroni-corrected). There was no significant decoding performance difference between the pSTS and premotor cortex.

There was also a significant interaction of task and feature (F = 6.702, *p* < 0.001, η²=0.024). When attention was directed to a specific feature within a task, the decoding performance peaked across the related task (female vs. male in the actor task, hand vs. foot in the effector task, and human vs. object in the target task). Notably, within the effector task, decoding for hand vs. foot surpassed both female vs. male (MD = 2.727, t = 3.956, *p* = 0.004, Bonferroni-corrected) and human vs. object (MD = 2.875, t = 4.171, *p* = 0.002, Bonferroni-corrected). Conversely, no significant distinction in feature decoding performances was observed within target and actor tasks.

Also, hemi*roi*task (F = 3.443, *p* = 0.011, η²=0.007) and roi*task*feature (F = 2.325, *p* = 0.021, η²=0.012) interactions were found significant. None of the other interactions were significant.

## Discussion

In this study, we investigated how top-down attention influences neural representations within the Action Observation Network, including the pSTS, parietal cortex, and premotor cortex. Using both univariate and multivariate (RSA and decoding) analyses, we examined how attending to different features of an observed action (effector, actor, or target) modulated neural responses in these ROIs. Across analyses, task manipulations influenced activity in all ROIs: RSA revealed significant correlations between the task model and neural patterns in multiple regions, and decoding analyses showed above-chance classification of task-relevant features within each ROI. Notably, the specific features that were most strongly represented differed across ROIs, indicating that each region was preferentially sensitive to particular task factors rather than responding uniformly to all.

To gain complementary insight into overall activation patterns, we additionally examined whole-brain univariate contrasts collapsed across tasks (e.g., Human > Object, Foot > Hand; see Supplementary Table 3), thresholded at *p* < 0.001 (uncorrected, *k* ≥ 50). The Human > Object contrast revealed modest activation in right occipitotemporal regions near the lateral occipitotemporal cortex (LOTC) and extrastriate body area (EBA), whereas the reverse contrast (Object > Human) did not yield comparable effects. The absence of strong EBA activation in the target condition is consistent with the nature of our stimuli: both Human and Object categories contained human figures—two interacting individuals versus one person interacting with an object—so body information was present in both. Classic EBA localizers typically contrast isolated bodies or body parts with non-body objects or tools (Downing et al. 2001; Peelen and Downing 2005), thereby isolating form-based body selectivity. In contrast, our manipulation emphasized interaction context and target identity rather than body presence per se, likely reducing sensitivity in body-selective visual areas. Similarly, the Foot > Hand contrast produced only subtle occipitotemporal activations, possibly reflecting small differences in effector-specific visual features rather than robust categorical effects. Together, these observations suggest that the relatively weak univariate contrasts reflect the conceptual structure of the task and the naturalistic complexity of the stimuli rather than methodological artifacts, confirming that the analysis pipeline performed as expected.

Next, we focused on the multivariate analyses, which provide higher sensitivity for detecting fine-grained representational patterns (Haynes 2015). RSA results revealed that all ROIs in the AON showed neural patterns aligned with the ongoing task: the task model, but not the feature models, demonstrated significant similarity to neural RDMs across all ROIs. This indicates a shared adaptability to attentional demands across the network. Notably, in the right premotor cortex, the correlation between the task model and neural patterns reached the noise ceiling, indicating that the variance in neural responses for this region was fully explained by the task model. In all other ROIs, none of the tested models reached the noise ceiling, suggesting that additional factors beyond the task model contribute to explaining their neural patterns. Consistent with Nastase et al. (2017), this pattern indicates that behavioral goals can reshape representational geometry in late-stage perceptual and motor-related regions, effectively disentangling population responses along task-relevant dimensions to optimize downstream read-out. To further examine these differences, we complemented the RSA with decoding analyses assessing how well specific task-relevant features could be discriminated within each ROI.

Decoding analysis provided a more detailed view of how attention influenced the ROIs. First, we conducted an ANOVA on accuracy-minus-chance values. There was no significant main effect of *task type*, suggesting that decoding performance was comparable across tasks. This is consistent with behavioral results showing no significant differences in response time or accuracy between task types, indicating similar cognitive demand. Likewise, no main effect of hemisphere was observed, indicating that left and right hemispheres performed similarly in action observation decoding. In contrast, significant main effects of ROI and feature were found, indicating that pSTS, parietal, and premotor regions differed in the overall amount of information they contained about the actions, and that some action features were decoded more accurately than others. A significant hemisphere × ROI × task type interaction indicated that the effect of task type on decoding varied across ROIs in each hemisphere. Similarly, the significant ROI × task type × feature interaction suggested that task type modulated the decoding of specific features differently across ROIs. Together, these interactions show that attention can have distinct effects on feature encoding depending on both the brain region and hemisphere.

Decoding results revealed distinct patterns of activity within each ROI, particularly in the active task compared to the passive task, extending previous findings on the specialization of AON regions (Stehr et al. 2021; Shahdloo et al. 2022; Orban et al. 2019). In the pSTS, classifications for female vs. male and hand vs. foot were not above chance in any task type. In contrast, human vs. object was successfully decoded in the actor task. In this classification, the “object” condition involved the actor manipulating an object, whereas the “human” condition involved the actor pushing another person, introducing a social context difference. The pSTS is a core node of the social brain (Dasgupta et al. 2017; Lahnakoski et al. 2012), and its sensitivity to social information may underlie this task-specific decoding success.

In the parietal cortex, hand vs. foot classification was successful under all three tasks in the left hemisphere and under two tasks in the right hemisphere. This aligns with prior work showing parietal encoding of effector information, including grip type and its interaction with goal (Errante et al. 2021). Across all features, decoding accuracies were higher in the parietal cortex than in other ROIs. This region has been implicated in encoding abstract action semantics (Gottlieb 2007) and in discriminating between action types (Orban et al. 2019), which may explain its robust performance across multiple features and tasks.

In the premotor ROI, decoding analyses revealed that in the left hemisphere, “hand vs. foot” was successfully decoded under the effector task, and “human vs. object” was successfully decoded under the target task. In the right hemisphere, “human vs. object” was decoded under the target task. These findings indicate a pronounced interaction between task context and the specific features represented within the premotor cortex. Classification of “female vs. male” was unsuccessful across all tasks, suggesting a lack of sensitivity to the actor factor in this region. This aligns with previous evidence that the premotor cortex primarily encodes the kinematics of actions (Binkofski and Buccino 2006; Takahashi et al. 2017), which are not directly altered by changes in the actor. Consistent with these decoding results, RSA showed that the right premotor cortex was the only region where the correlation between the task model and neural patterns reached the noise ceiling, suggesting a particularly strong representation of task-related distinctions in this region compared with other ROIs.

Wurm et al. (2016) contrasted the motor hypothesis, which posits that abstract action concepts are encoded in PMv, with the cognitive hypothesis, which attributes such representations to LOTC. They found that LOTC encoded both concrete and abstract actions across tasks, whereas PMv was restricted to concrete decoding in the explicit task; right inferior parietal cortex showed abstract decoding only in the explicit task. Our premotor findings align with this pattern: decoding was feature-specific and task-dependent, consistent with the cognitive hypothesis’ view of PMv as primarily encoding concrete information when task-relevant. However, in the passive session, we could still decode the target feature under the target task, suggesting that some form of abstraction may persist without explicit task demands. The parietal cortex in our study exhibited high decoding accuracies across features and tasks, consistent with Wurm et al.’s proposal of an “intermediate” role between PMv (concrete, task-dependent) and LOTC (concrete and abstract, task-independent). For pSTS, decoding was limited to the target feature in the target task; this weaker effect may partly reflect that our stimuli were not optimised to elicit strong biological motion responses in this region. Future studies using stimulus designs optimised for pSTS or extending the current paradigm to LOTC may help clarify how different AON nodes represent concrete and abstract action features.

Although top-down modulation of the AON may involve prefrontal contributions (e.g., during active task engagement), no prefrontal clusters survived whole-brain FWE correction in the active versus passive contrast. This absence likely reflects methodological factors, as our acquisition parameters and field of view were optimised for high coverage and resolution in occipito-temporal, parietal, and premotor cortices, with more limited coverage and SNR in anterior frontal regions. Thus, our findings do not exclude prefrontal involvement at subthreshold or distributed levels, but rather reflect the sensitivity of the current protocol. In addition, our design specifically targeted feature-based attention within the action observation domain—directing participants to attend to attributes such as goal, effector, or actor—rather than implementing a general attentional control task. While we cannot rule out contributions from broader attentional mechanisms (e.g., vigilance or task engagement), the localisation of effects to action-related ROIs and their systematic variation with attended feature suggest that our results primarily reflect feature-specific rather than generic attentional modulation. These conclusions therefore pertain to feature-based attentional modulation in action observation and may not generalise to other attention types.

### Limitations

Several factors should be considered when interpreting these findings:

*Task–motor confound*: In our design, only active task conditions required a motor response, whereas the passive condition did not. Although the GLM modelled the response phase separately from the video phase and decoding was based on the video-viewing phase, residual activity related to motor preparation and execution could still influence the BOLD signal, particularly in sensorimotor and premotor cortices. This may contribute to some of the active–passive differences observed. However, several aspects of our data suggest that motor responses are not the sole explanation: (i) we did not observe the strong lateralized premotor patterns expected from the fixed left/right thumb mapping, and (ii) task-specific effects also appeared in ROIs without direct motor involvement. Still, without a motor-matched control condition (e.g., passive viewing with intermittent button presses), we cannot entirely disentangle attentional from motor contributions.

*Stimulus diversity*: The stimulus set comprised eight videos depicting a single type of action. While the resulting activation patterns align with previous action observation research, the limited range of actions constrains the generalizability of the conclusions to other action types.

*Control over low-level visual features*: As the videos were designed to approximate real-life scenarios, perfect control of low-level features (e.g., actor clothing, start/end posture differences) was not feasible. Nonetheless, representational similarity analysis showed no meaningful correspondence between the low-level visual model and the neural RDMs, suggesting minimal influence of these factors.

*Eye-movement measures*: We did not track participants’ gaze during stimulus presentation. Eye movements, particularly with dynamic stimuli, may introduce variability in neural responses (Schütz et al. 2011). Eye-tracking in future studies would allow assessment of this potential source of variability.

### Future directions

Building on the present findings, several lines of research could extend and refine our understanding of how task demands shape neural representations of observed actions.

First, future studies could broaden the stimulus set to include a wider range of actions, actors, and contexts. This would help test whether the task-specific effects observed here generalise beyond the specific action class examined. Incorporating more naturalistic video material, including recordings of real-world actors in complex environments (Pekçetin et al. 2023; Maselli et al. 2023), could increase ecological validity while still allowing sufficient experimental control to disentangle perceptual from cognitive influences.

Second, systematic variation of task demands could clarify how different cognitive goals modulate activity across the Action Observation Network and related regions. For example, directly comparing tasks that emphasise different features of an action (e.g., movement kinematics vs. actor identity vs. action goal) could reveal distinct neural signatures and help to map the functional specialisation of individual nodes. Combining such designs with methods that better separate attentional from motor contributions (e.g., motor-matched passive conditions, response counterbalancing) would address some of the interpretational limitations of the current work.

Also, future research could explicitly examine the interaction between bottom-up sensory input and top-down cognitive modulation in action perception. This could be approached by jointly modelling low-level visual features, high-level conceptual information, and task demands in representational similarity or encoding models. Such an approach may also benefit from richer behavioural measures such as eye tracking to better relate neural patterns to viewing strategies during dynamic action observation.

Finally, methodological refinements such as optimising coverage for prefrontal cortex or increasing spatial resolution could help test whether frontal areas contribute to the modulation of action representations, a possibility not fully addressed here due to acquisition constraints.

## Conclusion

In this study, we examined how task demands modulate action perception within the AON. We found that the type of task influenced neural activity across multiple AON regions, with each ROI showing distinct changes in its representational patterns depending on the specific task requirements. Multivariate analyses revealed that these effects varied across regions in ways that aligned with their sensitivity to particular action features (e.g., movement kinematics, action goals, or actor identity). These findings indicate that top-down attentional modulation alters the neural coding of observed actions in a region-specific manner, with each area reflecting its characteristic pattern of feature encoding.

## Supplementary Information

Below is the link to the electronic supplementary material.

Supplementary Material 1

## Data Availability

The data will be available from the corresponding author upon request. The analysis scripts and the experiment videos can be accessed through OSF: https://osf.io/j4gc3/.
