# Supplementary material for: Top-down modulation of visual action perception: distinct task effects in the action observation network
Source: Brain Struct Funct. 2025 Nov 10;230(9):175. doi: 10.1007/s00429-025-03042-z (PMC12602651; doi:10.1007/s00429-025-03042-z)
Supplement: Supplementary file 1 — Supplementary Material 1 [file 429_2025_3042_MOESM1_ESM.docx]

**Supplementary Material**

In the passive session, our videos effectively stimulated the AON, which is evident from the activation of key regions associated with action observation. At a significance threshold of familywise error (FWE) corrected p<0.05, early visual areas including V1, V2, V3; the regions in the occipito-temporal cortex including the MT cluster and pSTS; and the PGp in the PPC were activated (Figure 6, top). At an uncorrected p<0.001 threshold, additional activations surfaced in the posterior parietal cortex (putative human anterior intraparietal sulcus (phAIP), PGp, PGa, PFt, PFpop, PFm, PFcm, PF) (Caspers et al., 2006) as well as in premotor cortex, V7, ventral intraparietal sulcus (VIPS), and BA2 region. These regions were illustrated by the white borders in both Figure 6 and Figure 7.

In the active session, contrasting all movies against rest (regardless of task) at a significance threshold of FWE corrected p<0.05 yielded similar activation patterns to the passive session while showcasing extended activation areas, notably within the posterior parietal cortex, premotor, and frontal regions. These supplementary activation regions include dorsal intraparietal sulcus anterior region (DIPSA), dorsal intraparietal sulcus medial region (DIPSM), phAIP (extended) (Orban, 2016), BA2 (extended), BA3, BA4, and premotor areas (Figure 6, bottom).


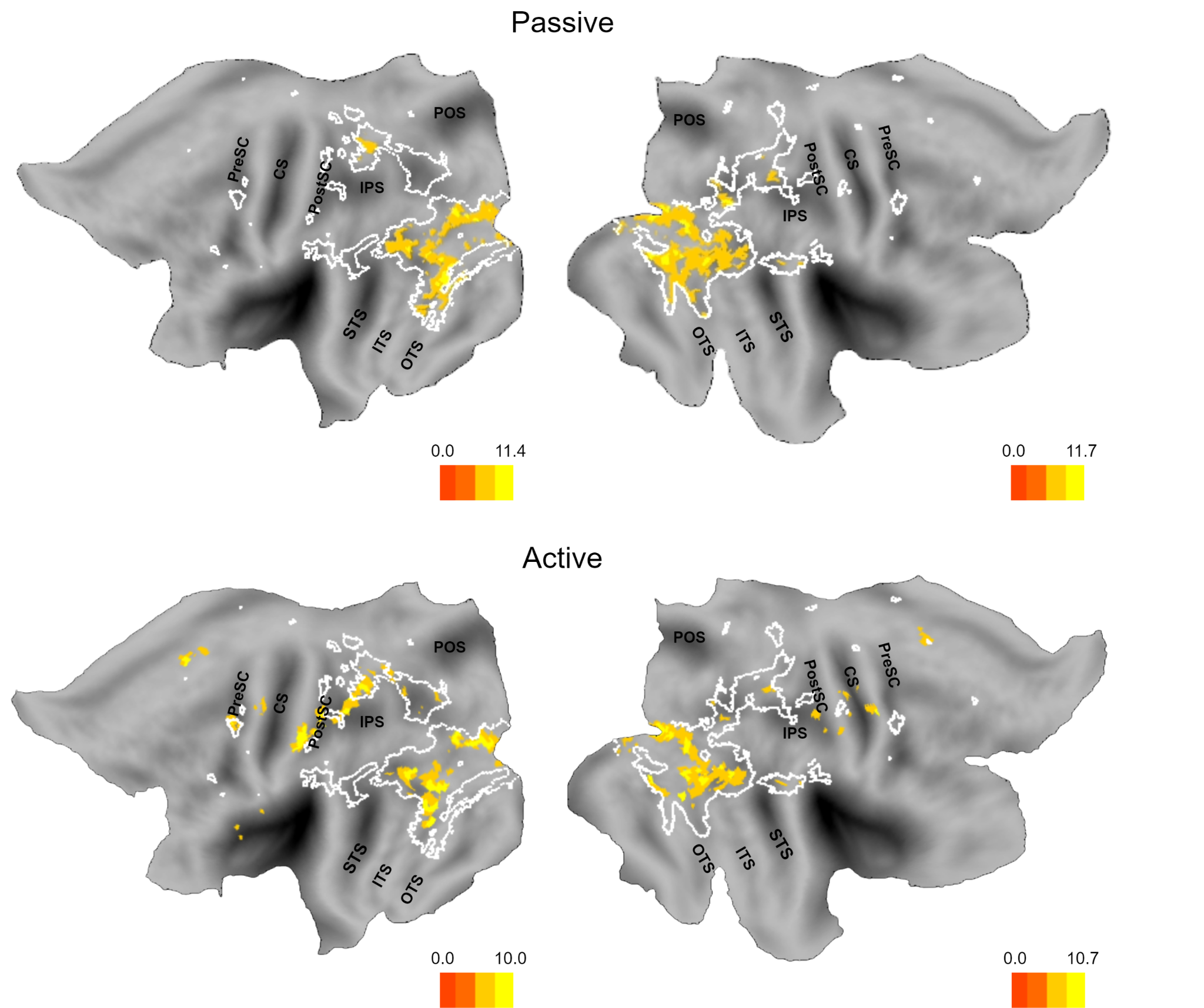


**Supplementary Figure 1.** Activation maps of “all videos minus rest” contrast from passive session (top) and active session (bottom). The results are adjusted for significance at p<0.05 with FWE correction. The white borders are from the contrast between the “all videos and the rest” condition from the passive session at p<0.001, uncorrected. Maps are visualized on the flat map using Caret5 software. Landmarks are indicated in black: PreSC (pre-central sulcus), CS (Central sulcus), PostCS (post-central sulcus), IPS (intra-parietal sulcus), POS (parieto-occipital sulcus), STS (superior temporal sulcus), ITS (inferior temporal sulcus), OTS (occipito-temporal sulcus)

Next, we visualized the activation maps for each task individually. The resulting activation patterns resemble those observed in the active session's "all tasks minus rest" condition, primarily localizing to regions associated with action observation (Figure 7).

Parietal region activity varies slightly across tasks. Notably, DIPSA is active in all tasks, while DIPSM is active in effector and target tasks within the left hemisphere. In the right hemisphere, DIPSA is active for the actor task. On the other hand, DIPSA and DIPSM show overlapping activity at the border between the two regions for effector and target tasks in the right hemisphere. OP1 region is active only in the effector task within the right hemisphere. In terms of the motor activation area derived from Ferri et al. (2015), the left hemisphere displayed activity during all tasks, while it is inactive in the right hemisphere. Also, in the left hemisphere, we observed activity in premotor dorsal (PMd), premotor medial (PMm), and premotor ventral (PMd) regions. In the right hemisphere, premotor activity was less pronounced. We only observed activity in PMm for the actor and target tasks. In the lateral occipitotemporal cortex, all three tasks activate areas on the middle temporal gyrus (MTG), MT+, and occipito-temporal sulcus (OTS), with very similar activation patterns for both hemispheres.

**
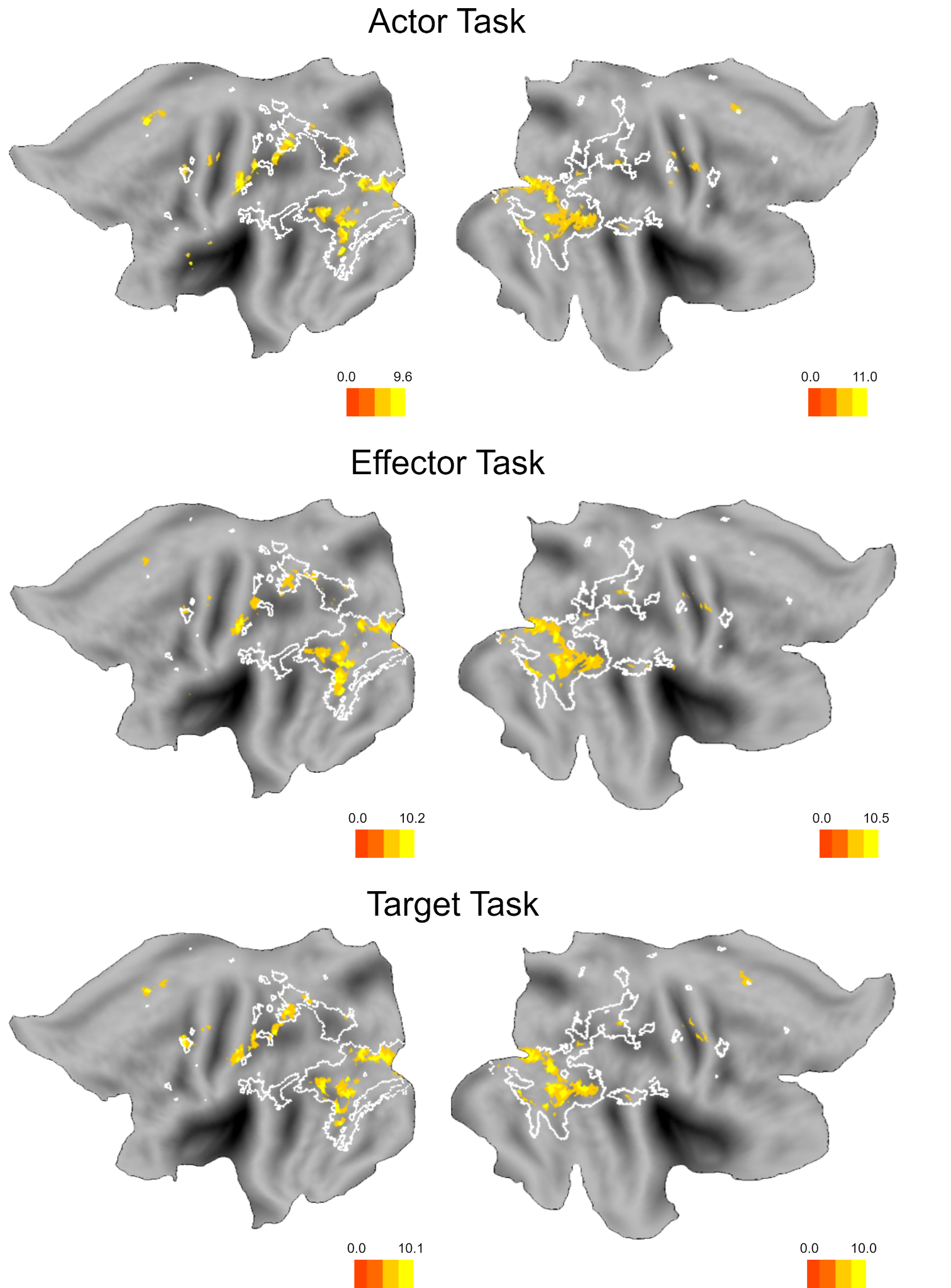
**

**Supplementary Figure 2.**  Activation maps of all videos minus rest contrast from the actor task (top), the effector task (middle) and the target task (bottom). The results are adjusted for significance at p<0.05 with FWE correction. The white borders are from the contrast between all videos and the rest condition from the passive session at p<0.001, uncorrected

**Supplementary Table 1**. Decoding accuracy minus chance calues and statistical significance (p-values and FDR-corrected p-values) for contrasts of interest (female vs. male, hand vs. feet, human vs. object) across brain regions of interest (left/right pSTS, left/right parietal, left/right premotor) for the active session.

| **Decoding Type** | **Mean Value** | **p value** | **p value - corrected** |
| --- | --- | --- | --- |
| left,psts,actor,hand vs feet | 0.4519400351 | 0.3332 | 0.51408 |
| left,psts,actor,human vs object | 1.06784612 | 0.1578 | 0.3156 |
| left,psts,actor,female vs male | -1.051311728 | 0.889 | 0.889 |
| left,psts,effector,hand vs feet | 1.070601852 | 0.1327 | 0.2756076923 |
| left,psts,effector,human vs object | -0.1846340388 | 0.595 | 0.714 |
| left,psts,effector,female vs male | -0.1198743386 | 0.5724 | 0.714 |
| left,psts,target,hand vs feet | 1.631393298 | 0.0792 | 0.1944 |
| left,psts,target,human vs object | 1.730599647 | 0.0218 | 0.06224210526 |
| left,psts,target,female vs male | -0.3789131393 | 0.6203 | 0.7281782609 |
| right,psts,actor,hand vs feet | 1.813271605 | 0.039 | 0.1002857143 |
| right,psts,actor,human vs object | 2.4085097 | 0.0126 | 0.04644 |
| right,psts,actor,female vs male | -0.2066798942 | 0.5861 | 0.714 |
| right,psts,effector,hand vs feet | 1.355820106 | 0.0912 | 0.2141217391 |
| right,psts,effector,human vs object | 0.2962411817 | 0.3585 | 0.5289081081 |
| right,psts,effector,female vs male | -0.5979938272 | 0.7122 | 0.7540941176 |
| right,psts,target,hand vs feet | 0.6117724868 | 0.2902 | 0.4748727273 |
| right,psts,target,human vs object | 1.36133157 | 0.1185 | 0.25596 |
| right,psts,target,female vs male | -0.4850088183 | 0.7076 | 0.7540941176 |
| left,parietal,actor,hand vs feet | 5.020943562 | 0.0002 | 0.00216 |
| left,parietal,actor,human vs object | 2.602788801 | 0.0129 | 0.04644 |
| left,parietal,actor,female vs male | 5.567956349 | 0 | 0 |
| left,parietal,effector,hand vs feet | 5.606536596 | 0.0001 | 0.00135 |
| left,parietal,effector,human vs object | 0.8170745151 | 0.1982 | 0.369062069 |
| left,parietal,effector,female vs male | 2.058531746 | 0.0117 | 0.04644 |
| left,parietal,target,hand vs feet | 2.724041005 | 0.0087 | 0.03915 |
| left,parietal,target,human vs object | 3.530092593 | 0.0025 | 0.016875 |
| left,parietal,target,female vs male | 0.4409171076 | 0.3288 | 0.51408 |
| right,parietal,actor,hand vs feet | 2.64412478 | 0.0056 | 0.02749090909 |
| right,parietal,actor,human vs object | 1.08162478 | 0.1749 | 0.3373071429 |
| right,parietal,actor,female vs male | 3.243496473 | 0.0005 | 0.0045 |
| right,parietal,effector,hand vs feet | 6.53659612 | 0 | 0 |
| right,parietal,effector,human vs object | 2.079199736 | 0.0245 | 0.06615 |
| right,parietal,effector,female vs male | 0.8584104939 | 0.2223 | 0.3872322581 |
| right,parietal,target,hand vs feet | 1.828428131 | 0.0189 | 0.06162352941 |
| right,parietal,target,human vs object | 4.13497575 | 0 | 0 |
| right,parietal,target,female vs male | 0.08404982359 | 0.4723 | 0.6539538462 |
| left,premotor,actor,hand vs feet | -0.02342372133 | 0.5036 | 0.67986 |
| left,premotor,actor,human vs object | 0.2163249559 | 0.4127 | 0.5864684211 |
| left,premotor,actor,female vs male | -0.8032958554 | 0.7268 | 0.7547538462 |
| left,premotor,effector,hand vs feet | 2.998236332 | 0.003 | 0.018 |
| left,premotor,effector,human vs object | -0.4367835097 | 0.6661 | 0.7493625 |
| left,premotor,effector,female vs male | 1.317239859 | 0.104 | 0.234 |
| left,premotor,target,hand vs feet | 0.3127755733 | 0.3624 | 0.5289081081 |
| left,premotor,target,human vs object | 3.608630952 | 0.0007 | 0.0054 |
| left,premotor,target,female vs male | 0.6200396826 | 0.2673 | 0.45106875 |
| right,premotor,actor,hand vs feet | 1.974481922 | 0.0219 | 0.06224210526 |
| right,premotor,actor,human vs object | -0.1322751323 | 0.5643 | 0.714 |
| right,premotor,actor,female vs male | 0.7743606702 | 0.2118 | 0.38124 |
| right,premotor,effector,hand vs feet | 1.90696649 | 0.0194 | 0.06162352941 |
| right,premotor,effector,human vs object | -0.3472222222 | 0.6388 | 0.7339404255 |
| right,premotor,effector,female vs male | -0.4023368606 | 0.6891 | 0.7540941176 |
| right,premotor,target,hand vs feet | -0.2934854498 | 0.5837 | 0.714 |
| right,premotor,target,human vs object | 2.110890653 | 0.0048 | 0.02592 |
| right,premotor,target,female vs male | -0.9286816578 | 0.8576 | 0.8737811321 |

**Supplementary Table 2.** Decoding accuracy minus chance calues and statistical significance (p-values and FDR-corrected p-values) for contrasts of interest (female vs. male, hand vs. feet, human vs. object) across brain regions of interest (left/right pSTS, left/right parietal, left/right premotor) for the passive session.

| **Decoding Type** | **Mean Value** | **p value** | **p value - corrected** |
| --- | --- | --- | --- |
| left,psts,female vs male | 0 | 0.518 | 0.666 |
| left,psts,hand vs feet | 0.4726080247 | 0.2503 | 0.4095818182 |
| left,psts,human vs object | 0.675154321 | 0.1719 | 0.3438 |
| right,psts,female vs male | -0.5594135803 | 0.8436 | 0.8449 |
| right,psts,hand vs feet | -0.009645061741 | 0.5113 | 0.666 |
| right,psts,human vs object | 1.50462963 | 0.0531 | 0.1998 |
| left,parietal,female vs male | 0.9645061729 | 0.1241 | 0.3191142857 |
| left,parietal,hand vs feet | 4.311342593 | 0 | 0 |
| left,parietal,human vs object | 1.215277778 | 0.0555 | 0.1998 |
| right,parietal,female vs male | -0.6847993827 | 0.8449 | 0.8449 |
| right,parietal,hand vs feet | 3.308256173 | 0.0005 | 0.0045 |
| right,parietal,human vs object | 1.215277778 | 0.1088 | 0.3191142857 |
| left,premotor,female vs male | 0.2700617284 | 0.3566 | 0.5349 |
| left,premotor,hand vs feet | 0.8101851852 | 0.2175 | 0.3915 |
| left,premotor,human vs object | 2.411265432 | 0.0057 | 0.0342 |
| right,premotor,female vs male | -0.3086419753 | 0.6378 | 0.76536 |
| right,premotor,hand vs feet | -0.3858024691 | 0.7075 | 0.7959375 |
| right,premotor,human vs object | 0.9355709877 | 0.1436 | 0.3231 |

**Supplementary Table 3.** Group-level univariate results collapsed across tasks, thresholded at *p* < 0.001 (uncorrected) with a cluster extent threshold *k* ≥ 50 voxels. Peak MNI coordinates are derived from the second-level SPM analysis, and anatomical labels were assigned using the AAL3 atlas (Rolls et al., 2020).

| **Contrast** | **Peak (x, y, z)** | **AAL3 region(s)** | **Cluster size (k)** |
| --- | --- | --- | --- |
| Female > Male | 38, –16, 49 | Precentral_R | 85 |
| Male > Female | –36, –18, 49 | Precentral_L | 118 |
| Foot > Hand | –36, –28, 62; –40, –70, 4; 42, –66, 6 | Postcentral_L / Occipital_Mid / Temporal_Mid | 53 / 115 / 77 |
| Hand > Foot | 36, –18, 46 | Precentral_R | 88 |
| Human > Object | 38, –24, 59; 50, –76, 12 | Precentral_R / Temporal_Mid_R | 132 / 73 |
| Object > Human | –42, –22, 62 | Precentral_L | 81 |
